# Mitochondrial fusion is a therapeutic vulnerability of acute myeloid leukemia

**DOI:** 10.1038/s41375-023-01835-x

**Published:** 2023-02-04

**Authors:** Clement Larrue, Sarah Mouche, Shan Lin, Federico Simonetta, Nastassja K. Scheidegger, Laury Poulain, Rudy Birsen, Jean-Emmanuel Sarry, Kimberly Stegmaier, Jerome Tamburini

**Affiliations:** 1 Translational Research Center for Hemato-Oncology, Faculty of Medicine, University of Geneva, Geneva, Switzerland; 2 Swiss Cancer Center Leman, Lausanne, Switzerland; 3 Department of Pediatric Oncology, Dana-Farber Cancer Institute and Boston Children’s Hospital, Boston, MA USA; 4 The Broad Institute of MIT and Harvard, Cambridge, MA USA; 5 Cancer Research Centre of Toulouse, UMR1037 Inserm, UMR5077 CNRS, Université de Toulouse 3 Paul Sabatier, Equipe Labellisée LIGUE 2018, 31037 Toulouse, France

**Keywords:** Acute myeloid leukaemia, Cancer metabolism

## Abstract

Mitochondrial metabolism recently emerged as a critical dependency in acute myeloid leukemia (AML). The shape of mitochondria is tightly regulated by dynamin GTPase proteins, which drive opposing fusion and fission forces to consistently adapt bioenergetics to the cellular context. Here, we showed that targeting mitochondrial fusion was a new vulnerability of AML cells, when assayed in patient-derived xenograft (PDX) models. Genetic depletion of mitofusin 2 (MFN2) or optic atrophy 1 (OPA1) or pharmacological inhibition of OPA1 (MYLS22) blocked mitochondrial fusion and had significant anti-leukemic activity, while having limited impact on normal hematopoietic cells ex vivo and in vivo. Mechanistically, inhibition of mitochondrial fusion disrupted mitochondrial respiration and reactive oxygen species production, leading to cell cycle arrest at the G_0_/G_1_ transition. These results nominate the inhibition of mitochondrial fusion as a promising therapeutic approach for AML.

## Introduction

Acute myeloid leukemia (AML) is a bone marrow-derived hematological cancer characterized by the expansion of immature myeloid cells with blocked differentiation and increased proliferative capacity [1]. While the overall cure rate of this disease remains low, especially among the elderly, precision therapies targeting specific AML vulnerabilities have recently changed treatment paradigms [2]. In particular, the Bcl-2 inhibitor venetoclax, which targets mitochondrial anti-apoptotic mechanisms, was shown to improve AML patient survival when combined with cytarabine or 5-azacytidine [3–6], suggesting mitochondria as a pertinent therapeutic target in AML.

Initially recognized for their key function in cellular bioenergetics, mitochondria have been implicated in various aspects of cell biology including apoptotic cell death and the generation of reactive oxygen species (ROS), metabolites and intermediates for biosynthetic metabolism [7]. Following the discovery that cancer cells engage in lactic acid fermentation rather than mitochondrial oxidation in the presence of oxygen (Warburg effect), numerous mitochondrial functions were found to be deregulated to fuel cancer cells [8], with implication in metabolic switches and resistance to chemotherapy of leukemic cells [9–11]. Interestingly, proteogenomic profiling on a large cohort of patients with AML revealed a specific subtype characterized by high expression of mitochondrial proteins and stronger complex I-dependent respiration, lower remission rate and survival and high sensitivity to venetoclax ex vivo [12]. Moreover, single-cell transcriptomics uncovered mitochondrial-driven adaptive resistance to venetoclax and chemotherapy, suggesting that targeting mitochondrial metabolism could improve therapeutic response in AML [13].

Mitochondria are constantly reshaped through the formation of cristae in the inner mitochondrial membrane (IMM), and a balance between fusion and fission involving the IMM and the outer mitochondrial membrane (OMM) [14]. This mitochondrial dynamics is tightly regulated by dynamin-related GTPase proteins. These include the mitofusins MFN1 and MFN2 and optic atrophy 1 (OPA1), involved in mitochondrial fusion, and dynamin-related protein 1 (DRP1), which regulates fission through interaction with OMM-located receptors including mitochondrial fusion factor (MFF) [14]. MFN1, MFN2 and OPA1 are the core components of the fusion process, tethering OMM (MFN1 and MFN2) and IMM (OPA1), although regulating other aspects of mitochondria morphology including cristae remodeling (OPA1) or endoplasmic reticulum-mitochondria contacts (MFN2) [15–17].

We disrupted mitochondrial dynamics balance in AML cell lines and patient-derived AML cells, and observed that depletion of pro-fusion (MFN1, MFN2, OPA1) effectors was a vulnerability of leukemic cells in vitro and in vivo. Differential gene expression (DGE) analysis revealed that mitochondrial fusion inhibition modulated cell cycle-related signatures, and investigations in various in vitro and in vivo AML models showed that this occurred mainly during the transition between G_0_ and G_1_ phases. Mechanistically, mitochondrial fusion inhibition depleted oxidative phosphorylation and ROS production, which in turn led to cell cycle inhibition. Finally, we showed that the small compound OPA1 inhibitor MYLS22 had anti-leukemic activity while sparing normal murine or human hematopoietic cells in vitro and in vivo. These results nominate the inhibition of mitochondrial fusion as a promising therapeutic approach for AML.

## Material and methods

### Cell lines and reagents

MOLM-14 and OCI-AML2 human AML cell lines were short-tandem repeats (STR) profiled periodically by PCR-single-locus-technology (Promega, PowerPlex21 PCR Kit, Eurofins Genomics, Luxembourg). Doxycycline (1 µg/mL) was from Sigma-Aldrich (Saint Louis, MO, USA). Mitotempo and MYLS22 were from MedChem Express (Monmouth Junction, NJ, USA).

### Constructs

We cloned shRNAs against *MFN1*, *MFN2*, *OPA1*, *MFF* and *DRP1*, as well as control shRNA into lentiviral vectors with mCherry- or GFP-tagged constitutive, and/or doxycycline (Dox)-inducible promoters. We cloned *MFN2* cDNA obtained through GeneArt gene synthesis technology (Thermo Fisher Scientific, Waltham, MA, USA) into the pSMAL (Addgene plasmid #161785) lentiviral expression vector using the Gateway cloning. These methods were reported [18, 19] and are detailed in the Supplementary Information.

### Electron microscopy

Samples were fixed in glutaraldehyde, and sections were prepared using ultracut E microtome (Reichert, Buffalo, NY, USA) and visualized under a Morgagni transmission electron microscope (FEI Company, Eindhoven, Netherlands), as reported [20] and detailed in the Supplementary Information.

### Hematopoietic cells from human subjects

De-identified patient-derived AML samples were obtained from PDX repository (Cancer Research Center of Toulouse, France) [9, 21]. A signed written informed consent for research use in accordance with the Declaration of Helsinki was obtained from patients. Human normal primary bone marrow CD34+ cells were purchased from ATCC. Cells were cultured in Iscove’s modified Dulbecco’s medium (IMDM) supplemented with 10% FBS and BIT (BSA 4 g/L, Insulin 5 µg/mL and Transferrin 60 µg/mL, all from Sigma-Aldrich, IMDM-BIT).

### Clonogenic assays

#### L-CFU assays

L-CFU assays were performed as previously described [22]. Briefly, AML cells were seeded at 10^5^/mL in H4230 medium (StemCell Technologies, Vancouver, Canada) supplemented with 10% of IMDM-BIT containing 50 ng/mL FLT3 ligand, 10 ng/mL IL-6, 50 ng/mL SCF, 25 ng/mL TPO, 10 ng/mL IL-3, 10 ng/mL G-CSF (Peprotech, Rock Hill, NJ, USA) and 50 µM β-mercapto-ethanol (Sigma-Aldrich). At day 7, L-CFU (colony of >20 cells) were scored under an inverted microscope.

#### Normal hematopoietic progenitor clonogenic assays

Normal CD34+ hematopoietic cells were seeded at 10^4^/ml in MethoCult H4034 Optimum medium (StemCell Technologies). The erythroid burst-forming units (BFU-E) and granulocyte-macrophage colony-forming units (CFU-GM) were counted under an inverted microscope at day 10.

### Flow cytometry and cell sorting

Flow cytometry was done on a Cytoflex flow cytometer (Beckman Coulter, Brea, CA, USA). The references for dyes and antibodies are provided in the Supplementary Information. When appropriate, human AML cells were detected on the basis of hCD33/hCD45 staining and sorted on an Astrios cell sorter (Beckman Coulter).

#### DAPI labeling

DAPI is a fluorescent stain that binds to DNA A-T-rich regions that inefficiently pass through intact cell membranes and therefore preferentially stain dead cells [23]. Cells (0.5–1 × 10^6^ cells) were washed twice in PBS, and resuspended in PBS containing DAPI (Thermo-Fischer Scientific, Waltham, USA).

#### KI67 labeling

PDX AML cells (0.5–1 × 10^6^ cells) were fixed 15 min in 4% paraformaldehyde (PFA, Sigma-Aldrich) and permeabilized with cold methanol for at least 1 h. Next, cells were stained with anti-Ki-67 antibody (Becton Dickinson (BD) Biosciences, Franklin Lake, NJ, USA) for 1 h and resuspended in PBS containing DAPI or DRAQ7 (Thermo-Fischer Scientific).

#### CFSE labeling

Cells (0.5–5 × 10^6^ cells/mL) were added with 1 µL of CFSE (CellTrace, Invitrogen) stock solution for 1 mL. Then 50 mL of 10% FBS-supplemented culture medium was added, cells were pelleted, resuspended in HCM and analyzed.

### Patient-derived xenograft assays

All animal studies were conducted in accordance with the guidelines of the Association for Assessment and Accreditation of Laboratory Animal Care International and with approval of the local ethics committee (Geneva health department, authorization GE/123/19). Adult NOD/LtSz-SCID/IL-2Rγchain null (NSG) mice (6–8 weeks old) were treated with 20 mg/kg busulfan (Busilvex, Sigma-Aldrich) by intraperitoneal administration. Two days after treatment with busulfan, 2 × 10^6^ viable primary human AML cells from patients were injected in the tail vein. After 10 to 16 weeks, mice were sacrificed, and human AML cell engraftment was quantified by detection of viable human CD45+/CD33+ cells via flow cytometry. MYLS22 was solubilized in 10% DMSO, 40% PEG300, 5% Tween-80 and 45% PBS, and given by daily intraperitoneal injection at the dose of 30 mg/kg. Animals were randomly assigned into treatment groups and investigators were blinded when assessing the outcome.

### Gene expression profiling

RNA quality was evaluated with a Bioanalyzer 2100 (using an Agilent RNA6000 nano chip kit, Santa Clara, USA), and 100 ng of total RNA was reverse transcribed using the GeneChip WT Plus Reagent Kit according to the manufacturer’s instructions (Affymetrix, Thermo-Fisher Scientific). Raw fluorescence intensities were normalized and analyzed as detailed in the Supplementary Information.

### Immunofluorescence and mitochondria size measurement

Cells (10^5^) were incubated with 200 nM MitoTracker Deep Red dye (Thermo-Fischer Scientific) for 30 min, and transferred to 0.01% poly-L-lysine (Sigma-Aldrich) coated glass slides (Thermo-Fisher Scientific). Next, cells were fixed 15 min in 4% formaldehyde then cold 100% methanol and mounted in ProLongTM Gold antifade medium with DAPI (Invitrogen). Images were acquired using a Zeiss LSM 800 microscope with Airyscan.

### Bioenergetics analysis experiments

Oxygen consumption was measured using a Cell Mito Stress Test kit (Agilent Technologies) on a Seahorse XF96 extracellular flux analyzer as previously reported [24]. Briefly, 2 × 10^5^ cells per condition were seeded in 96-well XF96 well plates coated with Cell-Tak (Becton Dickinson), and loaded with serum-free unbuffered IMDM medium. After 1-h incubation at 37 °C without CO2, wells were successively injected with oligomycin (inhibitor of ATP synthase), carbonilcyanide p-triflouromethoxyphenylhydrazone (fccp, a decoupling agent that disrupts the mitochondrial membrane potential) and combination of rotenone and antimycin A (Ro/AA that inhibit mitochondrial complexes I and III, respectively).

### Statistics

Differences between the mean values obtained for the experimental groups were analyzed using the two-tailed Student’s *t* test (Welch’s correction), or paired t-test when appropriate. In comparisons involving more than two groups, we used analysis of variance (ANOVA). Statistical analyses were performed using Prism software 9.1.2 (GraphPad, San Diego, CA, USA). Vertical bars indicate standard deviations. *P < 0.05, **P < 0.01, ***P < 0.001.

## Results

### Mitochondrial fusion is an AML dependency

**Fig. 1:**
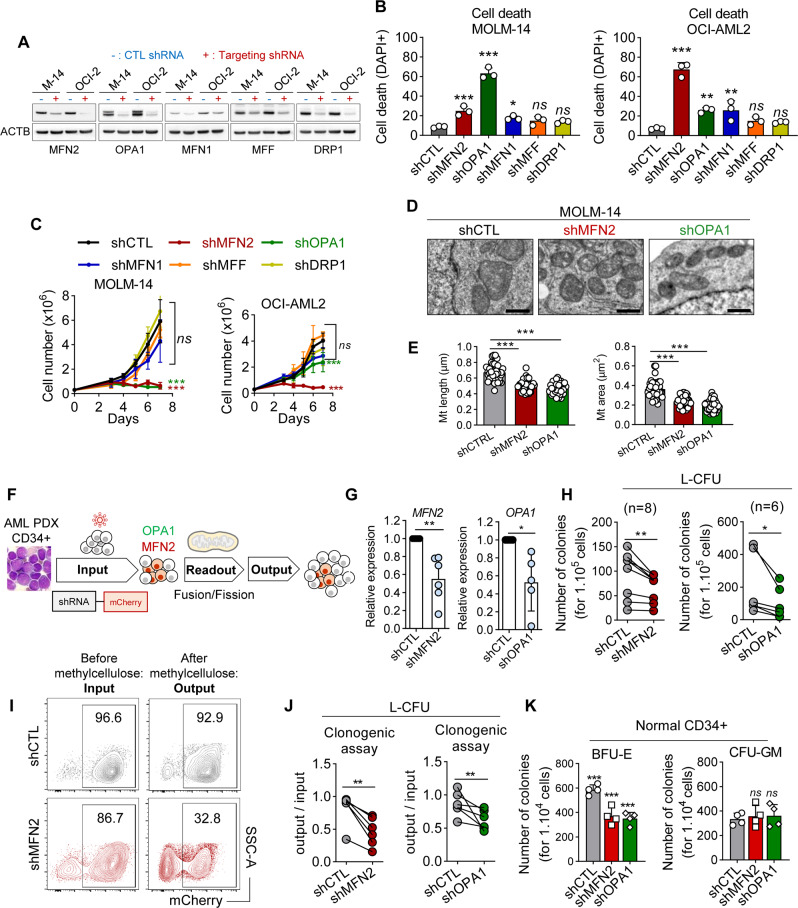
Mitochondrial fusion is an AML dependency. **A**–**E** MOLM-14 and OCI-AML2 cells were transduced with doxycycline (dox) inducible shRNA against MFN1, MFN2, OPA1, MFF or DRP1, or control (CTL) shRNA (*n* = 3). **A** Western blots performed 72 h after dox using anti-MFN1, -MFN2, -OPA1, -MFF, -DRP1 and -β-actin (ACTB) antibodies. **B** Cell death measured by DAPI staining 72 h after dox. **C** Cell proliferation measured by daily counting using trypan blue exclusion assay for the indicated time after dox. **D** Electron microscopy imaging at a 7100x magnification five days after dox. Scale bars = 5 μm. **E** Quantification of mitochondria (Mt) length (left panel) and area (right panel) in control, or MFN2- or OPA1-depleted MOLM-14 cells. Each dot represents Mt length in μm or area in μ^2^ (*n* = 30 cells, 5–50 Mt were measured in each cell). **F**–**K** PDX AML cells or normal immature CD34^+^ hematopoietic cells were transduced with mCherry-tagged shRNA targeting MFN2 or OPA1, or CTL shRNAs. In these mCherry^+^ transduced cells (input), we measured mitochondrial length (readout), performed ex vivo methylcellulose cultures to measure leukemia colony-forming units (L-CFUs), and evaluated the proportion of mCherry^+^ compared to mCherry^-^ cells (output). **G** Quantification of *MFN2* or *OPA1* transcripts relative to cells transduced with CTL shRNA in PDX AML cells (*n* = 6 different PDXs). **H** L-CFU assays with PDX AML samples scored after 7–10 days (shMFN2, *n* = 8 different PDXs; shOPA1, *n* = 6 different PDXs). **I**, **J** mCherry staining in human AML cells transduced by mCherry^+^ lentiviral vectors before or after methylcellulose culture. **I** Representative contour plots in CTL or MFN2 shRNA conditions. **J** Ratio of mCherry^+^ cells between the input (before methylcellulose) and the output (after methylcellulose) (*n* = 6). **K** Quantification of erythroid (BFU-E, left panel) and myeloid (CFU-GM, right panel) colonies from CD34^+^ cells after MFN2 or OPA1 depletion under an inverted microscope after 10 days of methylcellulose culture (*n* = 4). Vertical bars indicate standard deviations. ns not significant, *p < 0.05, **p < 0.01, ***p < 0.001.

To search for new mitochondrial vulnerabilities, we depleted pro-fusion (MFN1, MFN2, OPA1) and pro-fission (DRP1, MFF) effectors by doxycycline (Dox)-inducible shRNAs. In two AML cell lines (MOLM-14 and OCI-AML2), we observed by confocal microscopy imaging that depletion of fusion and fission effectors reduced and increased mitochondrial size, respectively (Fig. 1A and Supplementary Fig. S1A–D). We then observed that depletion of the pro-fusion proteins significantly induced leukemic cell death and inhibition of proliferation, in contrast to the inhibition of pro-fission effectors (Fig. 1B, C). However, we observed that MFN1 depletion was less toxic to leukemic cells compared to MFN2 or OPA1 depletion, possibly due to a lower efficacy of anti-MFN1 shRNA in our models (Supplementary Fig. S1A). We thus focused on MFN2 and OPA1 as models to investigate mitochondrial fusion, and confirmed that MFN2 or OPA1 depletion significantly shortened mitochondria in MOLM-14 and OCI-AML2 cells using electron microscopy (Fig. 1D, E).

Next, we depleted MFN2 or OPA1 using mCherry-tagged constitutive shRNAs in leukemic cells from patients with AML amplified in vivo using patient-derived xenograft (PDX) assays [25], or in normal human CD34^+^ hematopoietic progenitor cells (Fig. 1F). We significantly depleted MFN2 or OPA1 in PDX samples, which resulted in shorter mitochondria as measured by confocal imaging, and to a significant reduction of leukemia colony formation (L-CFU) [26] (Fig. 1G, H and Supplementary Fig. S1E, F). To evaluate the impact of MFN2 or OPA1 depletion during methylcellulose culture, we compared the proportion of leukemic cells efficiently transduced by mCherry^+^ lentiviral vectors before (referred to as input) and after (referred to as output) methylcellulose culture. We observed that MFN2 or OPA1 shRNA vectors significantly decreased the ratio between output and input compared to CTL shRNA (Fig. 1I, J). Notably, the output/input ratio was 0.86 in leukemic cells transduced with control (CTL) shRNA (*n* = 23), arguing that lentiviral transduction did not positively or negatively select AML cells in methylcellulose culture (Supplementary Fig. S1G). We observed the same anti-leukemic activity after MFN2 or OPA1 depletion using a second set of shRNAs in PDX samples (Supplementary Fig. S1H–J). Finally, we observed that MFN2 or OPA1 depletion in normal CD34^+^ cells decreased erythroid colony formation in vitro (BFU-E) while having no impact on myeloid colonies (CFU-GM) in methylcellulose culture [27] (Fig. 1K).

Together, these results showed that mitochondrial fusion is a new vulnerability in AML.

### Inhibition of mitochondrial fusion targets leukemia-initiating cells in vivo

**Fig. 2:**
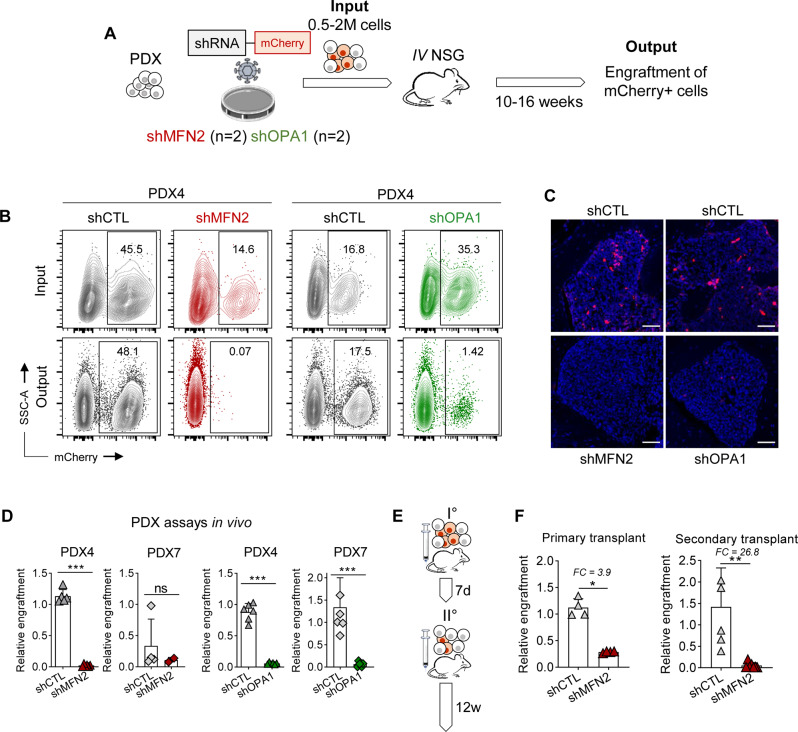
Inhibition of mitochondrial fusion targets leukemia-initiating cells in vivo. **A** PDX AML cells were transduced with mCherry-tagged shRNA targeting MFN2 or OPA1, or CTL shRNAs, then transplanted to recipient NSG mice. After 10–16 weeks, the engraftment of mCherry^+^ human AML cells was analyzed quantitatively by flow cytometry and qualitatively by confocal imaging on bone marrow tissue samples. **B** Representative contour plots for mCherry versus side-scatter-A (SSC-A) across the experimental conditions. **C** Representative confocal microscopy images at 20x magnification assessing the proportion of mCherry^+^ AML cells in CTL compared to MFN2- or OPA1-depleted conditions. Scale bars = 50 μm. **D** Relative engraftment defined as a ratio between the output (proportion of mCherry^+^ cells after 10–16 weeks) and the input (mCherry^+^ before transplantation). Results are plotted to compare MFN2 or OPA1 to the CTL conditions. Each dot indicates the relative engraftment in single mice. Two different PDX samples were transduced with CTL or anti-MFN2, or CTL or anti-OPA1 shRNAs and each of these four conditions was transplanted to five mice. **E**, **F** PDX AML cells were transduced with mCherry-tagged CTL or anti-MFN2 shRNAs, then transplanted to primary recipient NSG mice (*n* = 4 for each condition). Next, human AML cells were sorted after 7 days and transplanted to secondary recipient mice (*n* = 7 for each condition). Relative engraftment of mCherry^+^ cells was measured 12 weeks after transplant. **E** Schematic representation of the assay. **F** Relative engraftment after 12 weeks in shCTL and shMFN2 conditions. Fold-changes (FC) between the CTL and MFN2-depleted conditions are provided. Vertical bars indicate standard deviations. ns not significant, *p < 0.05, **p < 0.01, ***p < 0.001.

We investigated the impact of mitochondrial fusion inhibition in vivo in patient-derived xenograft (PDX) assays. In order to achieve high cell viability and improved lentiviral transduction rate, we first amplified primary samples from patients with AML (Table 1) by propagation to immunodeficient NOD/SCID gamma-null (NSG) mice [28]. Next, we transduced these PDX leukemic cells with lentivirus containing mCherry-tagged shRNAs against MFN2 and OPA1, or control (referred to as the input), and transplanted the cells to primary recipient NSG mice (Fig. 2A). Efficiently transduced human AML cells were tracked as hCD45^+^hCD33^+^mCherry^+^ among mice bone marrow samples (referred to as the output) 10–16 weeks after transplantation (Supplementary Fig. S2A).

**Table 1:** Characteristics of patient-derived AML samples.

Sample	Age	Sex	Leuko	Blast(%)	Origin	Genetics
PDX1	77.5	F	*198*	*76*	*PB*	*NPM, STAG2, DNMT3A*
PDX2	63.5	F	*163*	*98*	*PB*	*NPM1, TET2, DNMT3A*
PDX3	63.9	F	*128*	*92*	*PB*	*NPM1, DNMT3A, FLT3*
PDX4	63.9	M	*97*	*80*	*PB*	*DNMT3A, FLT3, NPM1*
PDX5	63.9	F	*280*	*33*	*PB*	*FLT3, DNMT3A, IDH2, SH2B3, NPM1*
PDX6	64.1	F	*176*	*90*	*PB*	*FLT3, DNMT3A, IDH2, NPM1*
PDX7	76.5	M	*88*	*93*	*PB*	*NRAS, NPM1, TET2*
PDX8	52.5	M	*55*	*91*	*BM*	*t(6;11), PTPN11*

Age in years.

*F* female, *M* male, *leuko* leukocytes (×10^9^/L), *blast(%)* percentage of blast cells in the sample, *origin* bone marrow (BM) or peripheral blood (PB), *genetics* pathogenic variants in genes relevant to myeloid neoplasms detected in diagnosis samples of patients with AML.

We observed that MFN2 or OPA1 depletion decreased the proportion of mCherry^+^ cells in the output compared to the input, while this population remained stable in the control (CTL) conditions (Fig. 2B). We corroborated these results by imaging mCherry in histological bone marrow samples, in which we observed very weak mCherry signal in MFN2- or OPA1-depleted conditions, while mCherry was unambiguously detected in the control conditions (Fig. 2C). We then defined the relative engraftment as the ratio of mCherry^+^ cells in the output *versus* the input conditions, which was significantly decreased in one out of two PDX after MFN2 depletion, and in two PDX after OPA1 depletion compared to the control (Fig. 2D). These results showed that inhibition of mitochondrial fusion effectors reduced leukemia initiation potential in PDX assays.

In a second set of experiments, leukemic cells were transduced ex vivo with CTL or MFN2 shRNAs, and transplanted to primary recipient NSG mice. In a pilot experiment, we observed that mCherry^+^ human leukemic cells were significantly depleted but still detected after one week in vivo, while this cell population was barely detected after twelve weeks (Supplementary Fig. S2B). We thus transplanted CTL or MFN2-depleted PDX AML cells to primary recipient mice, and sorted and transplanted an equal amount of human AML cells from mice bone marrow of these two experimental conditions to secondary recipient mice after one week (Fig. 2E). After 12 weeks, we observed a strong reduction of the relative engraftment in MFN2-depleted compared to CTL conditions in secondary recipient mice, suggesting that MFN2 inhibition reduced the serial transplantation capacity of leukemic cells in vivo.

Together this set of results show that mitochondrial fusion inhibition not only decreased leukemia propagation, but also reduced the self-renewal capacities of leukemic cells in vivo.

### Mitochondrial fusion inhibition regulates cell cycle at the G_0_/G_1_ transition

**Fig. 3:**
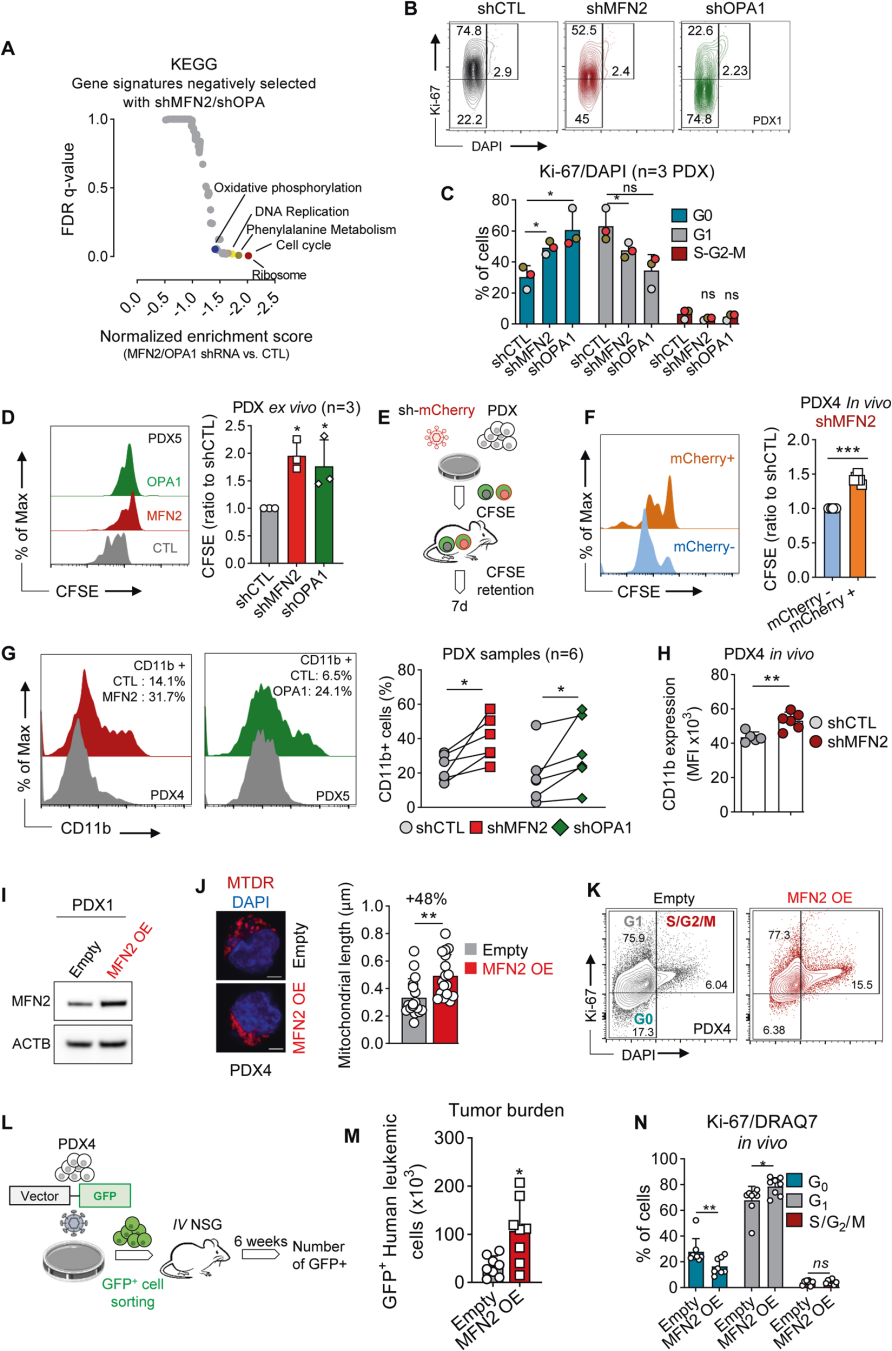
Mitochondrial fusion inhibition regulates cell cycle at the G_0_/G_1_ transition. **A** DGE analysis between CTL and MFN2- or OPA1-depleted PDX cells. Results are plotted as normalized enrichment score for KEGG signatures versus false discovery rate (FDR) q-value. **B**–**D** PDX AML cells were transduced with mCherry-tagged shRNA targeting MFN2 or OPA1, or CTL shRNAs and cultured in methylcellulose for 7–10 days before Ki67/DAPI staining (*n* = 3). **B** Representative contour plots of Ki67 versus DAPI. **C** Quantification of G_0_, G_1_ and S/G_2_/M cell cycle phases. **D** Cells were incubated in vitro in methylcellulose with 1 µL CFSE. Left panel: histograms of CFSE intensity versus cell number. Right panel: CFSE mean fluorescence intensity (MFI) quantification relative to the CTL condition (*n* = 3). **E** PDX AML cells transduced with mCherry^+^ anti-MFN2 shRNA were labeled ex vivo with CFSE, and propagated to NSG mice for 7 days (*n* = 7). **F** CSFE retention was quantified in mCherry^+^ (efficiently transduced) *versus* mCherry^-^ (non-transduced controls) cells. **G** PDX AML samples were transduced with mCherry-expressing control, anti-MFN2 or anti-OPA1 shRNAs (*n* = 6). Left panel: representative univariate flow cytometry histograms of CD11b expression among mCherry^+^ cells. The proportion of CD11b-positive cells is provided for each experimental condition. Right panel: proportion of CD11b^+^ cells. Results are plotted to compare CTL to MFN2 or OPA1 shRNA conditions. **H** PDX samples were transduced ex vivo with CTL or anti-MFN2 shRNA and transplanted to NSG mice. CD11b staining was quantified among efficiently-transduced mCherry^+^ cells 10–16 weeks after transplant (PDX4, *n* = 6 mice per group). **I**–**K** PDX AML samples were transduced with a pSMAL GFP-tagged vector for MFN2 overexpression (OE), or with the empty vector. **I** Western blots for MFN2 and ACTB expression. **J** Confocal imaging using MitoTracker Deep Red (MTDR) and DAPI staining. Scale bar = 2 μm. Quantifications are provided on the right panel (*n* = 30 cells per condition). **K** Flow cytometry contour plots of Ki67 *versus* DAPI in control or MFN2 OE PDX cells. **L**–**N** PDX4 AML cells were transduced with GFP^+^ empty or MFN2-OE vectors. Next, GFP+ cells were sorted and transplanted to NSG mice. AML tumor burden and cell cycle were evaluated after 6 weeks (*n* = 8 mice per group). **L** Experiment overview. **M** Quantification of AML tumor burden using GFP. **N** Cycle analysis among GFP^+^ cells using Ki67 and DRAQ7 staining. Vertical bars indicate standard deviations. ns not significant, *p < 0.05, **p < 0.01, ***p < 0.001.

To investigate the mechanisms underlying the anti-leukemic activity of mitochondrial fusion inhibition, we performed differential gene expression (DGE) analysis in transcriptomes of PDX samples and human AML cell lines after inhibition of MFN2 or OPA1, and we observed that the most significantly depleted signatures were related to cell cycle regulation (Fig. 3A and Supplementary Fig. S3A–C). Accordingly, propidium iodine (PI) staining assays performed in AML cell lines revealed that depletion of MFN2 or OPA1 significantly increased and decreased the proportion of G_1_ and S/G_2_/M cells, respectively (Supplementary Fig. S3D, E).

Next, we analyzed cell cycle repartition of PDX samples ex vivo. We measured quiescent G_0_ (Ki67^-^/DAPI^-^), G_1_ (Ki67^+^/DAPI^-^) and S/G_2_/M (Ki67^+^/DAPI^+^) cell populations [29], and observed that MFN2 or OPA1 depletion increased and decreased the proportion of G_0_ and G_1_ cells, respectively (Fig. 3B, C). We further used carboxyfluorescein succinimidyl ester (CFSE) labeling [30, 31] to investigate AML cell proliferation in leukemic cells grown in methylcellulose, and we observed that MFN2 or OPA1 depletion significantly enhanced CFSE retention compared to the control, showing that mitochondrial fusion inhibition decreased AML cell proliferation ex vivo (Fig. 3D). We also investigated cell proliferation in vivo using CFSE. After lentiviral transduction of MFN2 shRNA and CFSE labeling ex vivo, patient-derived AML cells were injected into NSG mice, and CFSE retention was evaluated after 7 days (Fig. 3E). We observed an increased CFSE retention in the efficiently-transduced mCherry^+^ compared to non-transduced mCherry^-^ populations, showing that MFN2 depletion led to decreased cell proliferation in vivo (Fig. 3F). Together, these results show that inhibition of mitochondrial fusion decreased the proportion of cycling leukemic cells in vitro and in vivo.

AML are characterized by a blockade at various stages of myeloid differentiation due to a dysregulation of transcription factors regulating the differentiation of normal hematopoietic cells [32]. Efficient targeted AML therapies frequently release this differentiation block [33]. In MOLM-14 and OCI-AML2 cells, we observed an increased cell surface expression of CD11b and CD14 (attesting for myeloid differentiation) after depletion of MFN2 or OPA1, which contrasted with the very little expression of CD11b or CD14 in the control condition (Supplementary Fig. S3F, G). Next, we used CD11b for further investigation of myeloid differentiation in PDX AML cells, and observed that MFN2 or OPA1 depletion significantly increased CD11b expression compared to the control cells ex vivo (Fig. 3G). We then measured CD11b in PDX assays, and observed that MFN2 depletion significantly increased CD11b compared to the CTL in vivo (Fig. 3H). Collectively, these results showed that inhibition of mitochondrial fusion promotes myeloid differentiation in AML.

In order to mirror mitochondrial fusion knockdown experiments, we forced mitochondrial fusion by MFN2 overexpression (OE) in AML cells by a GFP-tagged pSMAL lentiviral vector, which increased mitochondrial length compared to the empty control vector (Fig. 3I, J). DGE analysis of MFN2 OE cells compared to control showed that the most prominently modified pathways were related to cell cycle, and also to mitochondrial metabolism (oxidative phosphorylation) (Supplementary Fig. S3H, I). We further observed that MFN2 OE enhanced the proportion of cycling Ki67 and DAPI double-positive cells, and decreased CFSE retention compared to the control in AML PDX cells ex vivo (Fig. 3K and Supplementary Fig. S3J). We next xenografted MFN2 OE AML cells into NSG mice, and followed-up disease propagation in vivo after 6 weeks (Fig. 3L). First, we observed that the proportion of GFP^+^ leukemic cells was significantly increased in mice transplanted with MFN2 OE cells compared to the control, showing that forced mitochondrial fusion increased leukemia burden in vivo (Fig. 3M). Moreover, MFN2-OE leukemic cells had an increased and decreased proportion of G_1_- and G_0_-phase cells, respectively, compared to the control condition (Fig. 3N).

Collectively, these results show that mitochondrial fusion regulates proliferation and cell cycle in AML.

### Mitochondrial fusion regulates cell cycle through ROS production in AML

**Fig. 4:**
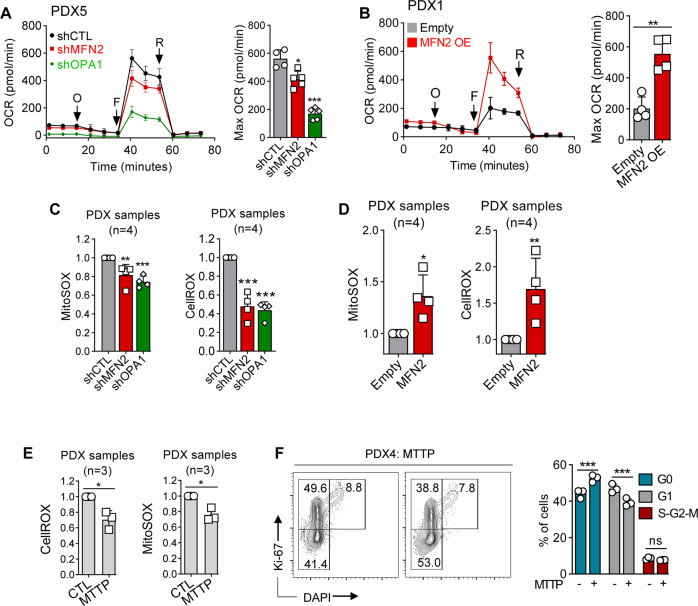
Mitochondrial fusion regulates cell cycle through ROS production in AML. **A**–**F** PDX AML cells were transduced with mCherry-tagged shRNAs against MFN2 or OPA1, or CTL shRNA, or with a GFP-tagged vector for MFN2 overexpression (OE), or with the empty vector. **A**, **B** Bioenergetic assays measuring oxygen consumption rate (OCR) dependent on time (in pmol/min). O: oligomycin; F: FCCP; R: rotenone/antimycin A. Left panels: OCR dependent on time. Right panels: quantification of maximal OCR after the addition of the uncoupling agent fccp. Experiments reported in panels **A** and **B** were done in PDX5 and PDX1 samples, respectively (*n* = 5 technical replicates for each assay). Quantification of total or mitochondrial ROS production using CellROX or MitoSOX staining, respectively, in shRNA (**C**) or OE (**D**) experiments. Results are presented relative to the control condition (*n* = 4). **E**, **F** Leukemic cells were incubated for 48 h with 50 nM mitotempo (MTTP) (*n* = 3). **E** Quantification of intracellular (CellROX dye, left panel) or mitochondrial (MitoSOX dye, right panel) ROS. Results are presented relative to the CTL condition. **F** Representative contour plots and quantification of cell-cycle phases using Ki67/DAPI staining. Vertical bars indicate standard deviations. ns not significant, *p < 0.05, **p < 0.01, ***p < 0.001.

Mitochondrial fusion is known to support increased oxidative phosphorylation (OxPhos) during cell proliferation [34], and we observed that mitochondrial fusion inhibition or activation depleted or promoted OxPhos gene expression signatures, respectively, in AML cells. Using bioenergetic assays, we further observed that MFN2 or OPA1 depletion significantly decreased the oxygen consumption rate (OCR) of leukemic cells, while MFN2 OE did the opposite (Fig. 4A, B and S4A, B). Mitochondrial electron transport chain is among the major endogenous sources of ROS [35]. Accordingly, we observed that total and mitochondrial ROS production, investigated by CellROX and MitoSOX staining, respectively, were inhibited or promoted upon mitochondrial fusion depletion or activation, respectively (Fig. 4C,D). These results showed that mitochondrial fusion regulated oxidative metabolism in AML cells.

We thus hypothesized that mitochondrial ROS content might regulate the transition between G_0_ and G_1_ phases of the cell cycle downstream of mitochondrial fusion. We used Mitotempo (MTTP), a mitochondria-targeted ROS scavenger compound [36], and observed that MTTP efficiently reduced total and mitochondrial ROS content in PDX AML cells (Fig. 4E). Moreover, MTTP decreased and increased the proportion of AML cells in G_1_ (Ki67^+^/DAPI^-^) and G_0_ (Ki67^−^/DAPI^−^) phases of the cell cycle, respectively (Fig. 4F). Similar results were observed with another ROS scavenger (Tempol), and with N-acetylcysteine in PDX AML cells (Supplementary Fig. S4C-D). As we observed an enrichment in mTORC1 signaling signature in MFN2-OE leukemic cells (Supplementary Fig. S3H), we hypothesized that mTORC1 could link ROS to cell cycle regulation downstream of mitochondrial fusion. Indeed, mTORC1 signaling was activated by MFN2 OE and inhibited after MFN2 or OPA1 suppression, while ROS depletion by MTTP inhibited mTORC1 signaling in AML cells (Supplementary Fig. S4E-J). Moreover, we incubated control or MFN2 OE AML cells with the prototypic mTORC1 inhibitor rapamycin [37], and observed that rapamycin decreased the G_1_/G_0_ ratio, and prevented the increased proportion of G_1_-phase cells induced by MFN2 OE (Supplementary Fig. S4K). These results suggest that depletion of ROS content after mitochondrial fusion suppression led to a blockade at the G_0_/G_1_ transition, which could have been mediated at least in part by mTORC1 signaling in AML.

### The small compound OPA1 inhibitor MYLS22 has anti-leukemic activity in vitro and in vivo

**Fig. 5:**
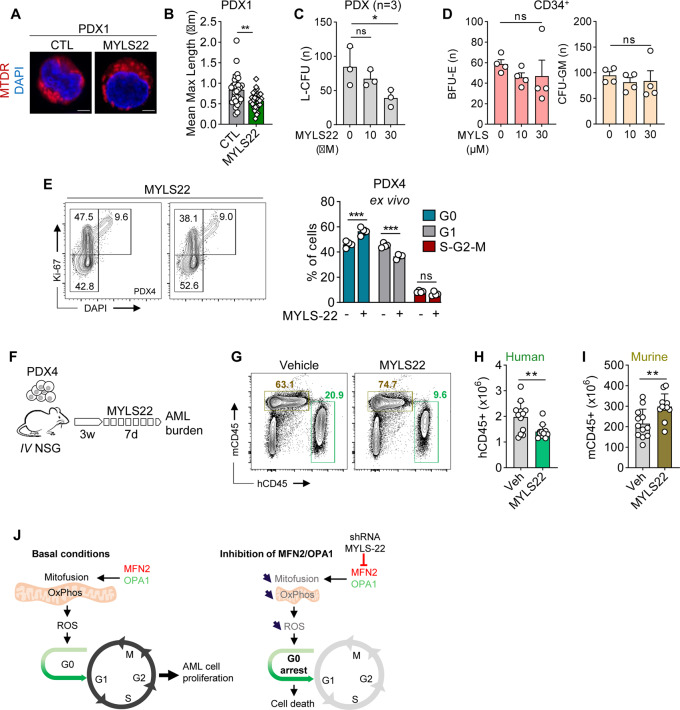
The small compound OPA1 inhibitor MYLS22 has anti-leukemic activity in vitro and in vivo. **A–E** PDX AML cells or normal CD34^+^ hematopoietic cells were incubated with vehicle or 10–30 μM of the small compound OPA1 inhibitor MYLS22 in methylcellulose. **A**, **B** Quantification of mitochondrial length using MTDR/DAPI staining and confocal imaging (63x objective. Scale bars = 2 μm) in PDX cells. **C** L-CFU assays on PDX AML cells after 7–10 days (*n* = 3). **D** Colony formation from normal human CD34^+^ hematopoietic progenitor cells after 10 days (*n* = 4). Left panel: BFU-E, right panel: CFU-GM. **E** Representative contour plots (left panel) and cell-cycle phase quantification (right panel) using Ki67/DAPI staining (*n* = 4). **F**–**I** Mice were treated with vehicle or 30 mg/kg MYLS22 by daily intraperitoneal injection during 7 days (*n* = 12 mice per arm). **G** Representative contour plots of hCD45 *versus* mCD45. **H** Quantification of hCD45^+^ human AML cells. **I** Quantification of mCD45^+^ murine hematopoietic cells. **J** MFN2 and OPA1 promote mitochondrial fusion, driving mitochondrial oxidative phosphorylation (OxPhos) and ROS production, which favor leukemic cells proliferation (left panel). After depletion of MFN2 or OPA1, or inhibition of OPA1 by the small compound MYLS22, inhibition of mitochondrial fusion results in ROS depletion and transition from G_1_ to G_0_ phase of cell cycle (right panel). Vertical bars indicate standard deviations. ns not significant, *p < 0.05, **p < 0.01, ***p < 0.001.

From the TCGA gene expression database, we observed that increased MFN2 expression could decreased the survival probability of patients with AML (Supplementary Fig. S5A). However, the only pharmacological inhibitor of mitochondrial fusion currently available is the small compound OPA1 inhibitor MYLS22 [38], which significantly reduced mitochondrial length and decreased ROS content in AML cells (Fig. 5A, B and Supplementary Fig. S5B). MYLS22 decreased the viability of MOLM-14 cells by 40% (IC_50_ 2.95 μM) while having no significant activity against OCI-AML2 cells (Supplementary Fig. S5C, D).

Interestingly, MYLS22 reduced the clonogenic growth of AML progenitors in L-CFU assays with an IC50 of 12.5 μM (Fig. 5C and Supplementary Fig. S5E). Notably, MYLS22 did not exhibit toxicity against normal erythroid and myeloid progenitor cells ex vivo (Fig. 5D and Supplementary Fig. S5E). Interestingly, MYLS22 increased and decreased the proportion of G_0_ (Ki67^-^/DAPI^-^) and G_1_ (Ki67^+^/DAPI^-^) PDX AML cells ex vivo, respectively (Fig. 5E). Finally, we observed that 7 days of in vivo treatment with MYLS22 significantly reduced tumor burden of leukemia-bearing mice, and increased and decreased the proportion of G_0_- and G_1_-phase human AML cells, respectively compared to the vehicle condition (Fig. 5F-H and Supplementary Fig. S5F). However, MYLS22 did not change the proportion of CD34^+^CD38^-^ leukemic cell population known to be enriched in leukemia-initiating cells [39] (Supplementary Fig. S5G). Notably, MYLS22 treatment had no significant impact on mice weight or on normal murine hematopoiesis in these assays (Fig. 5I and Supplementary Fig. S5H-I). These results confirm using a pharmacological approach that mitochondrial fusion represents a new vulnerability of AML.

Collectively, our results show that genetic or pharmacological inhibition of mitochondrial fusion disrupts oxidative metabolism, leading to an inhibition of cell cycle at the G_0_/G_1_ transition through the depletion of intracellular ROS content in AML (Fig. 5J).

## Discussion

Metabolic and mitochondrial adaptation represents emerging cancer hallmarks [7, 40]. We investigated the impact of targeted suppression of several key effectors of mitochondrial membrane fusion/fission, including MFN1, MFN2 and OPA1 (pro-fusion), and MFF and DRP1 (pro-fission), in AML PDXs and cell lines. Imbalanced mitochondrial membrane dynamics are commonly found in cancer, although limited data are available on the precise contribution of fusion and fission to cancer cell proliferation and metabolism [41]. In lung cancer, impaired fusion and enhanced fission caused by DRP1 activation of MFN2 inhibition fragment the mitochondrial network, which may facilitate mitosis [42]. Moreover, MFN2 overexpression suppress cancer progression, while low expression of this mitofusin is associated with poor prognosis in breast and lung cancer [43].

In AML, the DRP1 receptor fission, mitochondrial 1 (FIS1) is overexpressed and maintains the leukemic stem cell pool through mitophagy-mediated elimination of defective mitochondria [44]. Moreover, invalidation of caseinolytic mitochondrial matrix peptidase chaperone subunit B (CLPB), a mitochondrial protein involved in the maintenance of cristae structures through its interaction with OPA1 sensitize AML cells to venetoclax [16]. However, little is known on the role of mitochondrial membrane dynamics in AML. For this purpose, we established that repression of the effectors of mitochondrial fusion MFN2 and OPA1 had marked anti-leukemic effects, which were not observed after depletion of the fission factors DRP1 or MFF. We could hypothesize that this opposed contribution of fusion and fission could be due to cancer-specific metabolic reprogramming, as solid cancers and AML are generally dependent on glycolysis and mitochondrial oxidative metabolism, respectively [9, 41].

Using various AML PDX models, we showed that genetic or pharmacological inhibition of fusion effectors markedly impaired leukemia propagation in vivo. Moreover, suppression of MFN2 or OPA1 had a limited impact on the erythroid or myeloid differentiation of normal CD34+ human hematopoietic progenitor cells ex vivo, and the OPA1 inhibitor MYLS22 had no apparent toxicity against mouse hematopoietic cells in vivo. These results were in agreement with the established role of MFN2 in the maintenance of hematopoietic stem cells of lymphoid potential in mouse [45]. This differential effect between normal and malignant hematopoietic cells suggested that inhibition of mitochondrial fusion could represent a new therapeutic target in AML, especially because the depletion of erythroid precursors could be compensated by a transfusion management in clinical practice. Moreover, we might hypothesize that targeting mitochondrial fusion could synergize with other AML treatments such as cytarabine and/or venetoclax, as shown in other models of mitochondrial function disruption [9, 13, 19].

We further showed that mitochondrial fusion inhibition blocked the transition between G_0_ and G_1_ phases of the cell cycle ex vivo and in vivo. Mitochondrial dynamics are closely connected to the cell cycle, to allow appropriate distribution of fragmented mitochondria between daughter cells, but also to shut down mitochondrial oxidative metabolism in favor of biosynthesis pathways during mitosis [14, 46]. Strikingly, we observed that in our models, inhibition of mitochondrial fusion was the cause and not the consequence of cell cycle disruption. This finding was supported by mirror experiments in which forced mitochondrial fusion by MFN2 overexpression promoted G_0_ to G_1_ phase transition, which resulted to an increased leukemia burden in vivo in PDX models. In normal hematopoietic stem cells, blocking mitochondrial metabolism hinder differentiation and expand the stem cell pool [47, 48]. In contrast, we showed that suppression of mitochondrial fusion by MFN2 or OPA1 knockdown induced the differentiation of leukemic blasts, and that MFN2 depletion reduced both leukemia initiation and self-renewal in AML PDX assays in vivo, suggesting that the block in G_0_ phase induced by mitochondrial fusion inhibition did not result in a “stem-like” phenotype of quiescent cells in AML.

Mechanistically, MFN2 or OPA1 depletion disrupted mitochondrial respiration, while MFN2 overexpression increased oxygen consumption in leukemic cells, as observed in other models [34, 49]. Accordingly, activation or inhibition of mitochondrial fusion was followed by an increase or decrease in AML cells ROS content, respectively [50]. Moreover, depletion of mitochondrial ROS led to a decreased and increased proportion of G_0_ and G_1_ phase AML cells, respectively, suggesting that mitochondrial membrane dynamics regulate cell cycle through modulations of oxidative metabolism in AML cells. In support of this model, we observed that mTORC1, a multi-molecular complex integrating mitogenic and metabolic pathways to promote anabolism and known to control cell cycle in AML [51, 52] could regulate G_0_/G_1_ transition dependent on ROS content in AML. Interestingly, recent studies showed that ROS produced by mitochondrial respiration promote CDK2 phosphorylation [53], suggesting that ROS could have critical functions as signaling molecules to regulate cell cycle progression.

In conclusion, our study revealed that inhibition of mitochondrial fusion by MFN2 or OPA1 depletion induce potent anti-leukemic effects ex vivo and in vivo through an inhibition of cell cycle, unveiling a promising new therapeutic approach for AML. The significant anti-leukemic activity observed with the OPA1 inhibitor MYLS22 suggests that mitochondrial fusion could represent a future actionable target in AML.

## Supplementary information

all supplemental material

## Data Availability

The transcriptomic datasets generated and analyzed during the current study are available in Gene Expression Omnibus (GEO) database under the accession number GSE222169.
