# Supplementary material for: Mitochondrial fusion is a therapeutic vulnerability of acute myeloid leukemia
Source: Leukemia. 2023 Feb 4;37(4):765–75. doi: 10.1038/s41375-023-01835-x (PMC10079528; doi:10.1038/s41375-023-01835-x)
Supplement: Supplementary file 1 — all supplemental material [file 41375_2023_1835_MOESM1_ESM.pdf]

Supplementary Information

Mitochondrial fusion is a therapeutic vulnerability of acute myeloid leukemia

Larrue et al.

Table of Content:

- Supplementary Material and Methods, pages 1-10
- Supplementary References, page 11
- Supplementary Figures S1-S5 with legends, pages 12-20

Supplementary Material and Methods

Cell lines and reagents

MOLM-14 and OCI-AML2 human AML cell lines were identified by PCR-single-locus-technology (Promega, PowerPlex21 PCR Kit, Eurofins Genomics, Luxembourg). MOLM-14 harbors a FLT3-ITD mutation while OCI-AML2 carries a *DNMT3A* mutation, both mutations commonly found in AML [1]. AML cells were cultured in minimum essential medium (MEM)-α Glutamax (Life Technology, Carlsbad, CA, USA) supplemented with bovine serum albumin (BSA, Sigma-Aldrich, Saint Louis, MO, USA), 100 IU/mL penicillin and 100 µg/mL streptomycin (Life Technology) at 37°C under a 5% CO2 atmosphere. We also used HEK293T/17 cells cultured in 10% FCS-supplemented Dulbecco's modified Eagle medium (DMEM) Glutamax (Life Technology) for lentiviral production. Doxycycline was used at 1µg/mL and was from Sigma-Aldrich. MYLS22 was from MedChem Express (Monmouth Junction, NJ, USA).

Constructs

We obtained human *MFN2* ORF through GeneArt gene synthesis technology (Thermo Fisher Scientific, Waltham, MA, USA) and cloned them in pSMAL (Gift from John Dick & Peter van Galen, Addgene plasmid # 161785; http://n2t.net/addgene:161785 ; RRID:Addgene_161785) using the Gateway cloning. We cloned shRNAs against *MFN1*, *MFN2*, *OPA1*, *MFF* and *DRP1*, as well as control shRNA in the tet-pLKO-puro vector (Gift from Dmitri Wiederschain, Addgene plasmid # 21915; http://n2t.net/addgene:21915; RRID:Addgene_21915) allowing the conditional expression of hairpins by doxycycline. We also cloned shRNAs against *MFN2* and *OPA1*, as well as control shRNA in the pLKO.1 mCherry constitutive vector (Gift from Oskar Laur, Addgene plasmid # 128073; http://n2t.net/addgene:128073; RRID:Addgene_128073). Sequences of shRNAs are:

Control shRNA

- shCTL CAACAAGATGAAGAGCACCAA

First set:

- shMFN2-1 GCACTTTGTCACTGCCAAGAA
- shOPA1-1 CCGGACCTTAGTGAATATAAA
- shDRP1-1 GCTACTTTACTCCAACTTATT
- shMFF-1 TCAGTACGAAATGGAATATAC
- shMFN1-1 GCTCCCATTATGATTCCAATA

Second set:

- shMFN2-2 GCACTTTGTCACTGCCAAGAA
- shOPA1-2 GATTGCCCAAGCTCGAATATT

Lentiviral production

Briefly, we transfected HEK293T/17 cells with different plasmids together with the packaging plasmids pMD2.G (Gift from Didier Trono (Addgene plasmid # 12259; http://n2t.net/addgene:12259; RRID:Addgene_12259) and psPAX2 (Gift from Didier Trono (Addgene plasmid # 12260; http://n2t.net/addgene:12260 ; RRID:Addgene_12260) encoding lentiviral proteins using Lipofectamine 2000 Transfection Reagent (Thermo-Fischer Scientific). Twenty-four hours after cell transfection, medium was removed and opti-MEM culture medium (Life Technology, Carlsbad, CA, USA) was added. In order to transduce AML cell lines, HEK293T/17 culture supernatants containing lentiviral particles were harvested 72h after transduction, filtered, and stored at −80 °C. In order to transduce primary AML cells, collected supernatant were concentrated using PEG-it Virus Precipitation Solution according to manufacturer instructions (System Biosciences, Palo Alto, CA, USA), and stored at −80 °C.

Lentiviral infection

AML cell lines

On the day of transduction, 2×10^6 MOLM-14 or OCI-AML2 cells were resuspended in a medium containing 8µg/mL polybrene (Sigma-Aldrich, Cat# 107689) and 2 mL of thawed lentivirus-containing HEK293T/17 supernatant. Three days after infection, transduced cells were selected using 1µg/mL puromycin (LabForce, Muttenz, Switzerland) or 200µg/mL hygromycin (Invivogen, San Diego, CA, USA) dependent on the vector.

Human samples

Twelve-well plates were coated with 500µl retronectin (20 µg/mL in PBS) overnight at 4°C. Next, plates were blocked with 1 mL of 2% BSA PBS for 30 minutes at room temperature. Next, wells were washed once with PBS, 600 µL of concentrated lentiviral supernatant was added and plates were centrifuged (4000 rpm, 3h, 4°C) to favor virus adhesion to retronectin. After centrifugation, 0.4 mL of the viral supernatant was discarded and 1mL of the PCM culture medium containing 1-5×10^6 cells was added to each well. Plates were next centrifuged (1500 rpm, 10 minutes, RT) and incubated at 37°C for 24h.

Immunofluorescence and mitochondria size measurement

For Mitochondria staining, 10^5 cells were washed once in PBS, incubated in 50µL PBS with 200nM of the MitoTracker Deep Red dye (Thermo-Fischer Scientific) (30 minutes, 37°C, 5% CO2), and transferred to 0.01% poly-L-lysine (Sigma-Aldrich) coated glass slides (Thermo-Fisher Scientific). Next, cells were fixed in 4% formaldehyde for 10 minutes, washed in PBS, fixed in 100% methanol (-20°C, 5 minutes), washed (PBS and distilled water) and finally mounted in ProLongTM Gold antifade medium with DAPI (Invitrogen). Images were acquired using a Zeiss LSM 800 microscope with Airyscan. Length of mitochondria was determined with Image J software using the following macro: Image -> Type -> 8-bit; Image -> Adjust -> Auto Threshold -> default; Process -> Binary -> Make binary; Process -> Binary -> Convert to Mask; Process -> Binary -> Skeletonize; Analyze -> Skeleton -> Analyze Skeleton. For each cell, the average length was calculated.

Bioenergetic analysis assays

Oxygen consumption rate (OCR) was measured using a Seahorse XF96 extracellular flux analyzer (Seahorse Bioscience, North Billerica, MA, USA) and a Seahorse XF Cell Mito Stress Test Kit (Agilent Technologies, Santa Clara, CA, USA). Briefly, 2 × 10^5 cells were seeded in 96-well XF96 well plates coated with BD Cell-Tak (Becton Dickinson Biosciences, Franklin Lakes, NJ, USA). After 1h incubation at 37 °C without CO2, cells were transferred to the XF96 analyzer. Oligomycin (1µM) was added after 20 min, followed by FCCP (2µM) after 40 min and Antimycin A/Rotenone (1µM) after 59 min.

Clonogenic assays

L-CFU assays

L-CFU assays were performed as previously described [2]. Briefly, AML cells were seeded at 10^5/mL in H4230 medium (StemCell Technologies, Vancouver, Canada) supplemented with 10% PCM. At day 7, L-CFU (colony of > 20 cells) were scored under an inverted microscope.

Normal hematopoietic progenitor clonogenic assays

Normal CD34+ hematopoietic cells were purchased from StemCell Technologies. Cells were seeded at 10^4/ml in MethoCult H4034 Optimum medium (StemCell Technologies). The erythroid burst-forming units (BFU-E) and granulocyte-macrophage colony-forming units (CFU-GM) were counted under an inverted microscope at day 10.

Flow cytometry and cell sorting

Flow cytometry detection of dyes (TMRM, CellRox, Hoechst 33342 and MitoSox (from Thermo-Fischer Scientific), DAPI, CFSE and Pyronin Y (from Axonlab, Life Technologies and Sigma-Aldrich, respectively) was carried out using a Cytoflex flow cytometer (Beckman Coulter, Brea, CA, USA). The detection of cell surface markers was done on a Cytoflex flow cytometer using anti-hCD33 and -hCD45 antibodies, as reported [3]. The references for dyes and antibodies are provided. When appropriate, human AML cells were detected on the basis of hCD33/hCD45 staining and sorted on a Astrios cell sorter (Beckman Coulter). Intracellular phosphoflow for the detection of mTORC1 activity was carried out using anti-phospho-S6R (CST). Briefly, 0.5-1x10^6 PDX AML cells were fixed 15 min in 4% paraformaldehyde (Sigma-Aldrich) and permeabilized with cold methanol for at least one hour. Next, cells were stained with anti-phospho-S6 ribosomal protein antibody for 1 hour at RT and resuspended in PBS.

List of dyes

Reagent	Supplier	Reference
TMRM	Thermo Fisher Scientific	I34361
CellRox Deep Red	Thermo Fisher Scientific	C10422
MitoSox	Thermo Fisher Scientific	M36008
MitoTracker Deep Red	Thermo Fisher Scientific	M22426
DAPI	Axonlab	A4099.0
CellTrace CFSE Cell Proliferation Kit	Life Technologies	C34570
Pyronin Y	Sigma Aldrich	P9172
Hoechst 33342	Thermo Fisher Scientific	62249
DRAQ7	Thermo Fisher Scientific	D15106

List of antibodies

Antibody	Supplier	Reference
FITC Mouse Anti-Human CD45	Becton-Dickinson	555482
APC Mouse Anti-Human CD45	Becton-Dickinson	555485
CD33 PerCP-Cy™5.5	Becton-Dickinson	333146
PE Rat Anti-Mouse CD45	Becton-Dickinson	553081
FITC Mouse Anti-Human CD33	Becton-Dickinson	555626
ki67 BV650	Becton-Dickinson	563757
Ki67 FITC	Becton-Dickinson	556026

Patient-derived xenograft assays

All animal studies were conducted in accordance with the guidelines of the Association for Assessment and Accreditation of Laboratory Animal Care International and with approval of the local ethics committee (Geneva health department, authorization GE/123/19). Adult NSG mice (6–8 weeks old) were treated with 20 mg/kg busulfan (Busilvex, Sigma-Aldrich) by intraperitoneal administration. Two days after treatment with busulfan, $2x10^6$ viable primary human AML cells from patients were injected in the tail vein. After 10 to 16 weeks, mice were sacrificed, and human AML cell engraftment was quantified by detection of viable human CD45+/CD33+ cells via flow cytometry. After *ex vivo* manipulations (transduction with PLKO-mcherry or psMAL-GFP vectors), $0.5\text{-}2x10^6$ viable cells from these PDXs were injected into NSG mice as described above. At the end of experiments, mice were sacrificed, and human CD33/CD45 staining identified human AML cells from murine hematopoietic cells.

Cell cycle analysis

Propidium iodine

MOLM-14 or OCI-AML2 cells were fixed with 70% ethanol, and incubated for 2 hours in ice. Then cells were resuspended in FxCycle propidium iodine (PI)/RNase staining solution (Invitrogen) before analysis on a Cytoflex flow cytometer.

KI67 labelling

PDX AML cells (0.5-1x10^6 cells) were fixed in 4% paraformaldehyde (Sigma-Aldrich) (15 minutes, RT), washed twice in PBS and permeabilized with cold methanol (2h, on ice). Next, cells were washed twice in PBS, and stained with anti-Ki-67 antibody (Becton Dickinson) (1h, RT). Then, cells were washed twice in PBS, resuspended in PBS containing DAPI or DRAQ7 (Thermo-Fischer Scientific) and analysis were performed on a Cytoflex flow cytometer (Beckman Coulter).

PyroninY/Hoechst labelling

PDX AML samples (0.5-1x10^6 cells) were washed with PBS and then incubated (45 min, 37°C) with 20 g/mL Hoechst 33342 (Invitrogen) in Hanks balanced salt solution (HBSS) containing 10% FCS, 20mM Hepes and 50 µg/ml verapamil with pH=7.5. Next, 1µg/mL PyroninY (Sigma-Aldrich) was added, and cells were incubated (15 min, 37°C) before prompt processing for analysis (Cytoflex cytometer) or cell sorting (Astrios cytometer).

CFSE labelling

Cells were washed with PBS and then 1µL of CFSE (CellTrace, Invitrogen) stock solution was added to 1mL of cell culture at 0.5-5x10^6 cells/mL before incubation (30 minutes, 37°C) in the dark. Then 50 mL of 10% FBS-supplemented culture medium was added, cells were incubated (5 min, RT), and then pelleted by centrifugation and resuspended in PCM at 37°C.

Electron microscopy

We fixed 2x10^6 cells in 2% glutaraldehyde in 0.1M sodium phosphate buffer (pH 7.4) for 45 min, then post-fixed for 1.5 hours with 1% osmium tetraoxide and incubated for 12h in 2% uranyl acetate. Cells were then dehydrated by successive washes in a buffer with increasing ethanol concentration (70%, 90%, 100% and

100%), and successive incubation in 100% propylene oxyde, 50-50% propylene oxide/epon and epon. After polymerization, 80-90 nm sections were prepared using an ultracut E microtome (Reichert, Buffalo, NY, USA), stained with 2% uranyl acetate plus Reynold's lead citrate, and visualized under a Morgagni transmission electron microscope (FEI Company, Eindhoven, Netherlands). All reagents were from Sigma-Aldrich except uranyl acetate (Polysciences, Philadelphia, PA, USA).

Immunohistochemistry

Femurs and tibias of mice were fixed for 24h in 4% paraformaldehyde. Decalcification was carried out using 15% formic acid at 4°C for 4h, followed by a second fixation in 4% paraformaldehyde for 24h. Samples were paraffin embedded and then sliced using a ultracut E microtome (Reichert-Jung). Images were acquired and processed using the slide scanner and software Zeiss Axioscan.Z1 (Carl Zeiss AG, Oberkochen, Germany). Detection of human AML cells transduced with a mCherry-expressing lentiviral vector was done using a LSM700 confocal microscope (Zeiss, Stuttgart, Germany).

Gene expression profiling

Gene chip hybridization

RNA was extracted using a RNeasy Mini Kit (Qiagen, Redwood City, CA, USA) and quality was evaluated with a Bioanalyzer 2100 (using Agilent RNA6000 nano chip kit), and 100 ng of total RNA was reverse transcribed using the GeneChip® WT Plus Reagent Kit according to the manufacturer's instructions (Affymetrix, Thermo Fischer Scientific). Briefly, cDNA were hybridized to GeneChip® Clariom S Human (Affymetrix) at 45°C for 17 hours, then washed on the fluidic station FS450 (Affymetrix), and scanned using the GCS3000 7G (Thermo Fischer Scientific). Scanned images were then analyzed with Expression Console software (Affymetrix, Thermo Fischer Scientific) to obtain raw data (.cel files) and metrics for quality controls.

Data processing

Raw fluorescence intensity values were normalized using Robust Multiarray Average (RMA) algorithm in R to generate the normalized data matrix by performing background correction, quantile normalization and log2 transformation of raw fluorescence intensity values of each gene. All quality controls and statistics were performed using Partek® Genomics Suite software (Partek, St. Louis, MO, USA). Data were normalized using custom brainarray CDF files (v20 ENTREZG). To identify differentially expressed genes, we applied a classical analysis of variance (ANOVA) with a FDR permutation-base for each gene. We created a matrix with only the significant ANOVA site and performed Z-scoring of rows. Hierarchical clustering by Pearson's dissimilarity and average linkage and principal components analysis (PCA) were conducted in an unsupervised fashion to control for experimental bias or outlier samples.

Gene set enrichment analysis

We set a filter for those genes that displayed at least a ≥1,5 or ≤-1,5 fold difference in expression between groups and achieved an FDR of <0.05. Data were then interrogated for evidence of biologic pathway dysregulation using Gene set enrichment analysis (GSEA, Broad Institute). Enrichment rates were considered as significant for P-value <0.05 and FDR ≤0.1.

Quantitative PCR

Total RNA was prepared with GenElute Mammalian Total RNA miniprep (Sigma-Aldrich). cDNA was synthesized by Superscript II Reverse Transcriptase (Invitrogen, Waltham, MA, USA). Genes of interest were quantified using SYBR Green Power UP (Life Technologies). All reactions were carried out in a STEPone instrument (Applied Biosystems, Waltham, MA, USA). Results were quantified by the ΔCt method using *HYWAZ* or *P0* as standard nonvariable genes. List of primers is provided in the Supplemental methods section.

Western blots

Cells were lysed in NuPAGE™ LDS Sample Buffer supplemented with NuPAGE™ Sample Reducing Agent (Life Technologies) and heated for 5 min at 90°C. Then proteins were separated using 4–12% gradient

polyacrylamide SDS–PAGE gels (Life Technologies) and electrotransferred to 0.2 µm nitrocellulose membranes (GE Healthcare, Chicago, IL, USA). After blocking in Tris-buffered saline with 0.2% Tween and 5% bovine serum albumin, membranes were blotted overnight at 4 °C with the appropriate primary antibodies. Primary antibodies were detected using the appropriate horseradish peroxidase-conjugated secondary antibodies. Immunoreactive bands were visualized by enhanced chemiluminescence (PI32209; Thermo-Fisher Scientific) with a Pxi camera (Syngene, Cambridge, UK). Antibodies used are listed below.

Antibody	Supplier	Reference
MFN2	Cell Signaling Technology	11925S
OPA1	Cell Signaling Technology	80471
MFN1	Cell Signaling Technology	14739
MFF	Cell Signaling Technology	84580
DRP1	Cell Signaling Technology	5391
β-actin	Sigma-Aldrich	A2228

Statistics

Differences between the mean values obtained for the experimental groups were analyzed using the two-tailed Student’s t test (Welch’s correction), or paired t-test when appropriate. In comparisons involving more than two groups, we used analysis of variance (ANOVA). Statistical analyses were performed using Prism software 9.1.2 (GraphPad, San Diego, CA, USA). Vertical bars indicate standard deviations. *P<0.05, **P<0.01, ***P<0.001.

Supplementary References

1. Papaemmanuil, E. *et al.* Genomic Classification and Prognosis in Acute Myeloid Leukemia. *N. Engl. J. Med.* **374**, 2209–2221 (2016).
2. Tamburini, J. *et al.* Protein synthesis is resistant to rapamycin and constitutes a promising therapeutic target in acute myeloid leukemia. *Blood* **114**, 1618–1627 (2009).
3. Larrue, C. *et al.* Adrenomedullin-CALCRL axis controls relapse-initiating drug tolerant acute myeloid leukemia cells. *Nat Commun* **12**, 422 (2021).
4. Satija, R., Farrell, J. A., Gennert, D., Schier, A. F. & Regev, A. Spatial reconstruction of single-cell gene expression data. *Nat. Biotechnol.* **33**, 495–502 (2015).
5. Stuart, T. *et al.* Comprehensive Integration of Single-Cell Data. *Cell* **177**, 1888-1902.e21 (2019).

Figure S1

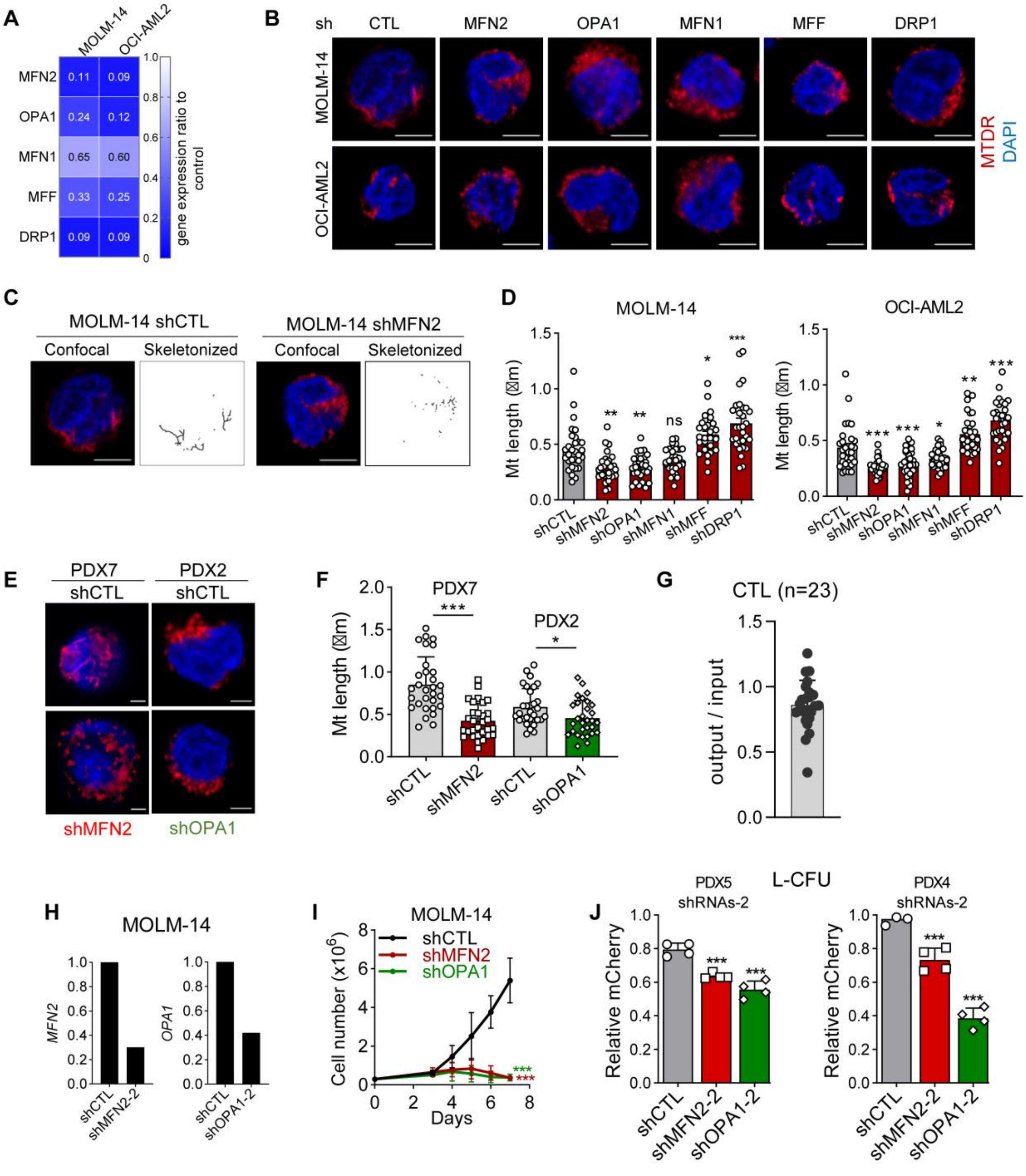

A
MOLM-14
OCI-AML2
MFN2 0.11 0.09
OPA1 0.24 0.12
MFN1 0.65 0.60
MFF 0.33 0.25
DRP1 0.09 0.09
gene expression ratio to control
B
sh CTL MFN2 OPA1 MFN1 MFF DRP1
MOLM-14
OCI-AML2
MTDR
DAPI
C
MOLM-14 shCTL
MOLM-14 shMFN2
Confocal
Skeletonized
D
MOLM-14
OCI-AML2
Mt length (⊠m)
shCTL shMFN2 shOPA1 shMFN1 shMFF shDRP1
E
PDX7 shCTL
PDX2 shCTL
shMFN2
shOPA1
F
PDX7
PDX2
Mt length (⊠m)
shCTL shMFN2 shCTL shOPA1
G
CTL (n=23)
output / input
H
MOLM-14
MFN2
OPA1
shCTL shMFN2-2
shCTL shOPA1-2
I
MOLM-14
shCTL
shMFN2
shOPA1
Cell number (x10^6)
Days
J
L-CFU
PDX5 shRNAs-2
PDX4 shRNAs-2
Relative mCherry
shCTL shMFN2-2 shOPA1-2

Figure S1. Mitochondrial fusion is an AML dependency. A-D. MOLM-14 and OCI-AML2 human AML cell lines were transduced with lentivirus expressing dox-inducible shRNAs targeting MFN1, MFN2, OPA1, MFF and DRP1, or a control (CTL) non-targeting shRNA. **A.** Quantification of *MFN1*, *MFN2*, *OPA1*, *MFF* and *DRP1* transcripts expression by quantitative PCR. Results are presented relative to the CTL condition using a heatmap format. **B.** Mitochondria and nuclei were stained with MitoTracker Deep Red (MTDR) and DAPI (blue), respectively, and cells were analyzed via confocal microscopy using a 63x objective. Scale bars = 5μm. **C.** Example of mitochondrial network length measurement after tracing mitochondria outlines ("skeletonized") using ImageJ software in MOLM-14 cells expressing control or anti-MFN2 shRNA. Images were processed using a 63x objective. Scale bars = 5μm. **D.** Quantification of mitochondria length across each experimental condition (n=30 cells per condition). **E-F.** PDX AML cells were transduced with mCherry-tagged shRNA targeting MFN2 or OPA1, or CTL shRNAs. **E.** Confocal imaging after MTDR and DAPI staining using a 63x objective. Scale bars = 5μm. **F.** Quantification of mitochondria length (n=30 cells per condition, n=2 PDX). **G.** Relative mCherry (ie. output/input ratio) in PDX AML cells transduced with lentiviral vectors expressing CTL shRNAs (n=23). **H-J.** MOLM-14 or PDX AML cells were transduced with a second set of mCherry-tagged shRNA targeting MFN2 or OPA1, or CTL. **H.** Quantification of *MFN2* and *OPA1* transcripts by quantitative PCR relative to the CTL condition in MOLM-14 cells. **I.** Cell proliferation assessed by daily cell counting in MOLM-14 cell line (n=3). **J.** Relative mCherry (output/input ratio) in two AML PDX after L-CFU assays (n=4 for each PDX). Vertical bars indicate standard deviations. ns: not significant, *p<0.05, **p<0.01, ***p<0.001.

Figure S2

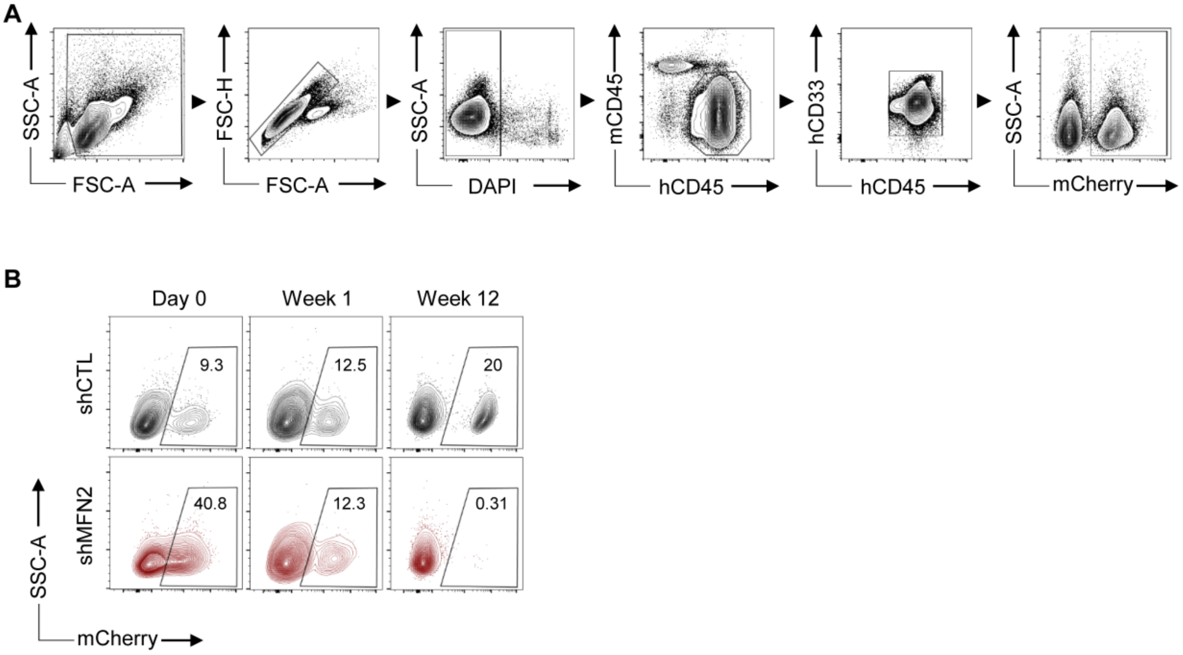

Figure S2. Inhibition of mitochondrial fusion targets leukemia-initiating cells *in vivo*. A. Flow cytometry gating strategy to identify human AML cells from mouse hematopoietic cells in PDX assays. Forward scatter area (FSC-A), side scatter area (SSC-A), anti-mouse CD45 (mCD45), anti-human CD45 (hCD45) and anti-human CD33 (hCD33) antibodies were used. **B.** PDX AML samples were transduced *ex vivo* with mCherry-tagged CTL or anti-MFN2 shRNA, and then xenografted to NSG mice. mCherry *versus* SSC-A contour plots are provided before (day 0) and one or twelve weeks after transplant.

Figure S3

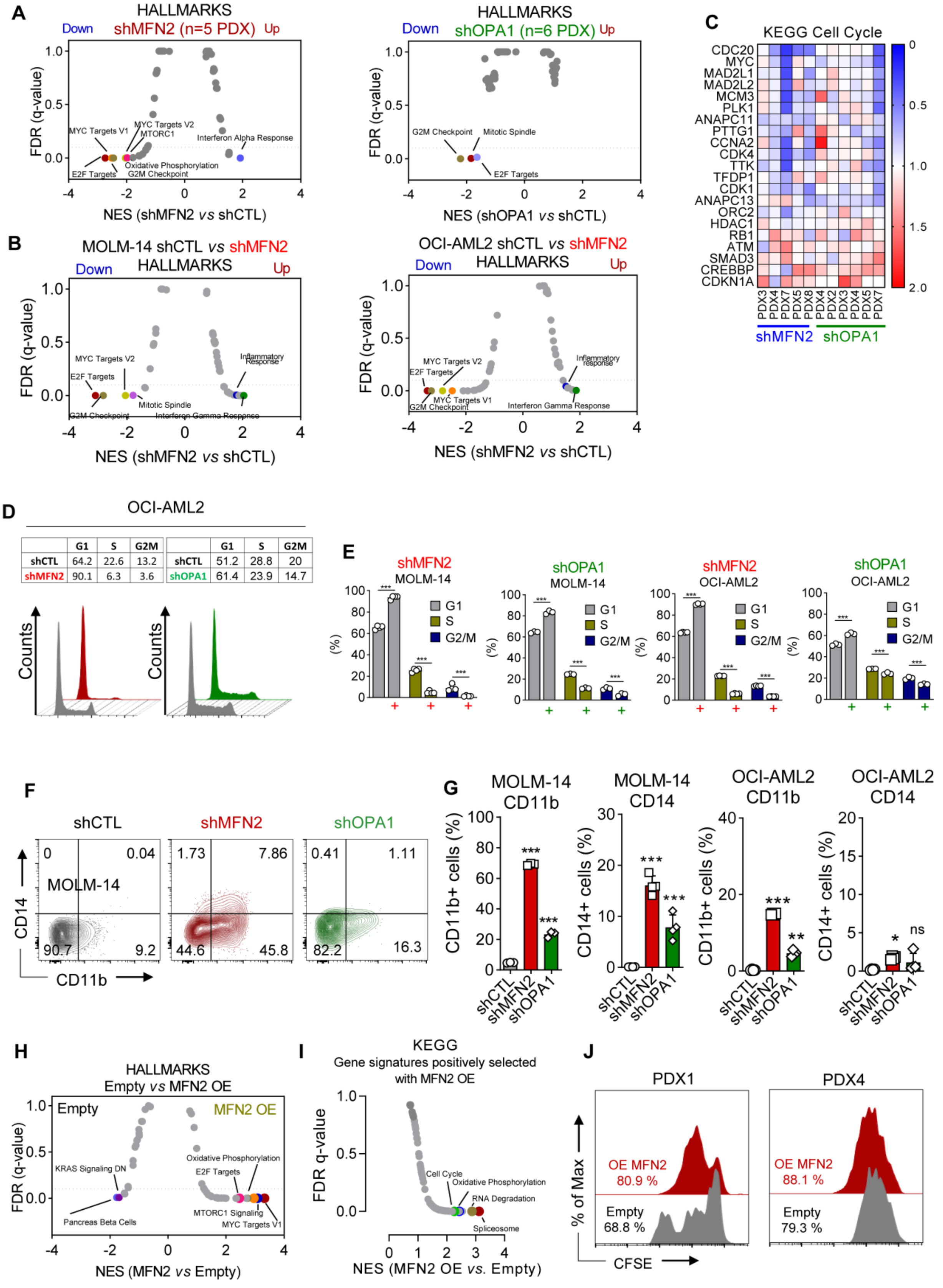

	G1	S	G2M
shCTL	64.2	22.6	13.2
shMFN2	90.1	6.3	3.6

	G1	S	G2M
shCTL	51.2	28.8	20
shOPA1	61.4	23.9	14.7

Figure S3. Mitochondrial fusion inhibition regulates cell cycle at the G_0/G_1 transition. A-B. DGE analysis in PDX AML cells (**A**, n=5-6) or in MOLM-14 and OCI-AML2 cells (**B**, n=3) after MFN2 or OPA1 depletion. Analysis of Hallmark gene sets are provided on a volcano plot format (false discovery rate (FDR) q-value versus normalized enrichment score (NES)), with a highlight of gene sets involved in cell cycle and proliferation. Gene sets downregulated in MFN2- or OPA1-depleted cells compared to CTL are indicated by DOWN. **C.** Enrichment in KEGG cell cycle signature in MFN2- or OPA1-depleted compared to CTL cells represented using heatmap. **D-E.** Propidium iodine (PI) staining in MOLM-14 and OCI-AML2 cells five days after induction of MFN2 or OPA1 by dox (n=4). **D.** Representative histograms. **E.** Relative quantification of G_1, S and G_2/M phases. **F-G.** MOLM-14 cells were induced for expression of CTL, anti-MFN2 or anti-OPA1 shRNAs by dox for 5 days. **F.** Representative contour plots of CD11b *versus* CD14 in MOLM-14. **G.** Quantification of CD11b and CD14 positive cells (n=3). **H-J.** DGE in PDX AML cells transduced with MFN2 OE or empty vector (n=3). **H-I.** Hallmark (**H**) and KEGG (**I**) gene sets plotted as normalized enrichment score (NES) *versus* false discovery rate (FDR) q-value. **J.** Univariate histogram of flow cytometry CFSE quantification in empty vector or MFN2-OE PDX cells. The proportion of cells shifted from the most intense peak are provided (n=2 PDX). Vertical bars indicate standard deviations. ns: not significant, *$p<0.05$, **$p<0.01$, ***$p<0.001$.

Figure S4

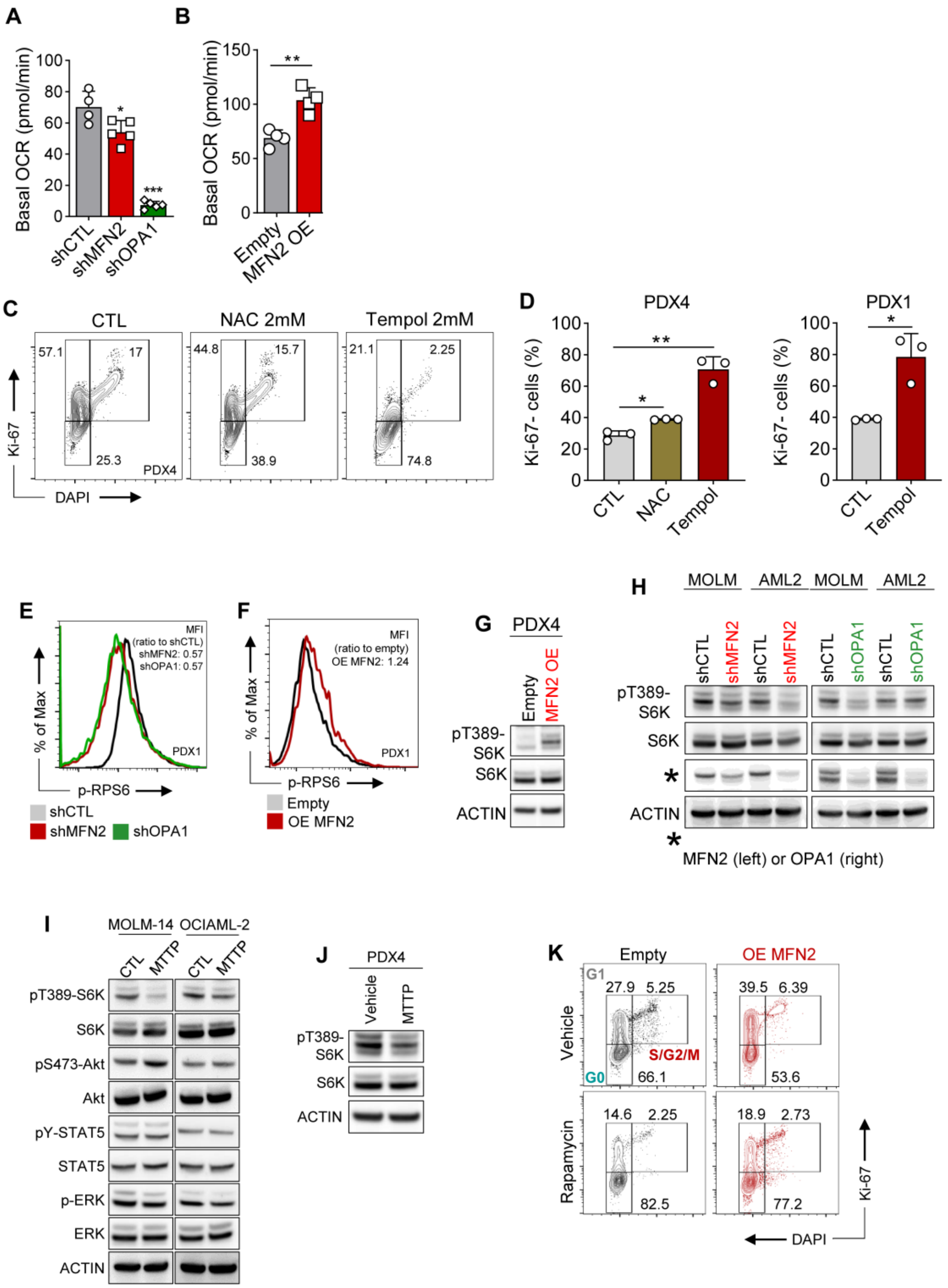
A
Basal OCR (pmol/min)
shCTL
shMFN2
shOPA1
B
Empty
MFN2 OE
C
CTL
NAC 2mM
Tempol 2mM
Ki-67
DAPI
PDX4
D
PDX4
PDX1
Ki-67- cells (%)
CTL
NAC
Tempol
E
MFI
(ratio to shCTL)
shMFN2: 0.57
shOPA1: 0.57
% of Max
p-RPS6
PDX1
shCTL
shMFN2
shOPA1
F
MFI
(ratio to empty)
OE MFN2: 1.24
Empty
OE MFN2
G
PDX4
pT389-S6K
S6K
ACTIN
H
MOLM
AML2
MFN2 (left) or OPA1 (right)
I
MOLM-14
OCIAML-2
MTTP
pS473-Akt
Akt
pY-STAT5
STAT5
p-ERK
ERK
J
Vehicle
K
OE MFN2
Rapamycin
G1
G0
S/G2/M

Figure S4. Mitochondrial fusion regulates cell cycle through ROS production in AML. A-B. Bioenergetic assays measuring OCR (in pmol/min) in PDX AML cells transduced with mCherry-tagged shRNAs against MFN2 or OPA1, or CTL shRNA (**A**) or with a GFP-tagged vector for MFN2 overexpression (OE), or with the empty vector (**B**). Experiments reported in panels **A.** and **B.** were done in PDX5 and PDX1 samples, respectively (n=5 technical replicates for each assay). **C-D.** PDX AML cells were treated *ex vivo* with vehicle, 2mM N-acetylcysteine (NAC) or 2mM tempol during 24h. Cell cycle was investigated using Ki67/DAPI staining. **C.** Representative panel. **D.** Evolution of the Ki67 negative population during NAC or tempol treatment in PDX4 and PDX1 samples. **E-H.** Leukemic cells were transduced with shRNA targeting MFN2 or OPA1, or control shRNA, or with MFN2-overexpressing (MFN2-OE) or control (empty) vectors. **E-F.** Representative univariate plots of p-RPS6 in PDX AML cells. **G-H.** Western blots using anti-phospho-P70S6k T389, -P70S6K, -MFN2, -OPA1 and –actin antibodies. **I-J.** Leukemic cells were incubated for 48h with 50nM mitotempo (MTTP). **I.** Western blots in MOLM-14 and OCI-AML2 cell lines using phospho-specific and total antibodies for p70S6K, Akt, STAT5 and ERK, and anti-actin antibody as loading marker. **J.** Western blots in PDX AML cells using anti-phospho-p70S6K, -p70S6K and –actin antibodies. **K.** Empty vector or MFN2-OE PDX AML cells were incubated for 48h with vehicle or 10nM rapamycin before Ki67 and DAPI staining. Contour plots of DAPI versus Ki67 are represented alongside with the percentage of G0, G1 and S/G2/M cells. Vertical bars indicate standard deviations. ns: not significant, *$p<0.05$, **$p<0.01$, ***$p<0.001$.

Figure S5

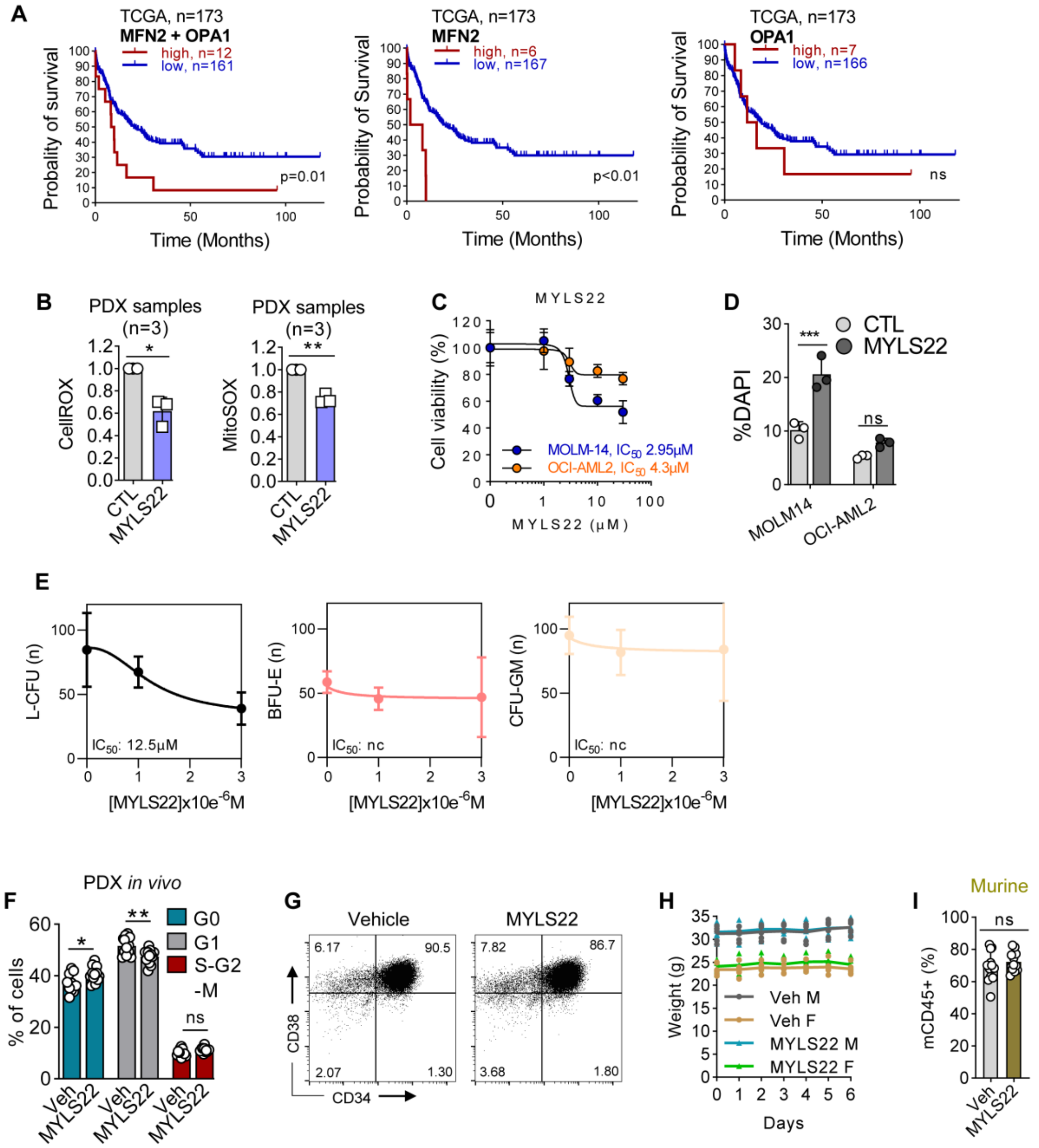
A
TCGA, n=173
MFN2 + OPA1
high, n=12
low, n=161
Probality of survival
p=0.01
Time (Months)
TCGA, n=173
MFN2
high, n=6
low, n=167
Probability of Survival
p<0.01
Time (Months)
TCGA, n=173
OPA1
high, n=7
low, n=166
Probability of Survival
ns
Time (Months)
B
PDX samples (n=3)
CellROX
CTL
MYLS22
PDX samples (n=3)
MitoSOX
C
MYLS22
Cell viability (%)
MOLM-14, IC50 2.95µM
OCI-AML2, IC50 4.3µM
MYLS22 (µM)
D
CTL
MYLS22
%DAPI
ns
MOLM14
OCI-AML2
E
L-CFU (n)
IC50: 12.5µM
[MYLS22]x10e-6M
BFU-E (n)
IC50: nc
[MYLS22]x10e-6M
CFU-GM (n)
IC50: nc
[MYLS22]x10e-6M
F
PDX in vivo
G0
G1
S-G2-M
% of cells
ns
Veh
MYLS22
G
Vehicle
MYLS22
6.17
90.5
2.07
1.30
7.82
86.7
3.68
1.80
CD38
CD34
H
Weight (g)
Veh M
Veh F
MYLS22 M
MYLS22 F
Days
I
Murine
mCD45+ (%)
ns
Veh
MYLS22

Figure S5. The small compound OPA1 inhibitor MYLS22 has anti-leukemic activity *in vitro* and *in vivo*. A. MFN2 expression correlates with survival in the TCGA AML database. We accessed the TCGA RNAseq data through the cBioPortal for cancer genomics online tool. MFN2 and/or OPA1 mRNA expression values were defined as high or low for z-scores > or < 2, respectively. The comparison of survival curves dependent on MFN2 or/and OPA1 high or low expression was done by the log-rank method using Prism software. **B.** PDX AML cells were incubated with vehicle or 30 μM of the small compound OPA1 inhibitor MYLS22 for 48h, and intracellular (CellROX dye, left panel) or mitochondrial (MitoSOX dye, right panel) ROS were measured. Results are presented relative to the CTL condition (n=3). **C-D.** MOLM-14 and OCI-AML2 cells were incubated with MYL22 as indicated, and cell viability (trypan blue exclusion assay, n=4, **C**) and cell death (DAPI staining, n=3, **D**) were measured after 48h. **E.** AML or normal CD34+ hematopoietic progenitor cells were incubated with vehicle (0) or 10-30μM MYLS22 during 7-10 days in methylcellulose-containing medium. A nonlinear regression curve was plotted from the results of colony formation number and IC_{50} values were calculated using Prism software. **F-I.** Mice were treated with vehicle or 30mg/kg MYLS22 by daily intraperitoneal injection during 7 days (n=12). **F.** The proportion of G_0 ($Ki67^-/DAPI^-$), G_1 ($Ki67^+/DAPI^-$) and $S/G_2/M$ ($Ki67^+/DAPI^+$) cells was measured by flow cytometry. **G.** Human CD34/CD38 staining in bone marrow samples from mice. **H.** Evolution of mice weight in grams (g) in PDX AML experiments. M: male; F: female. **I.** Percentage of murine bone marrow hematopoietic cells. Vertical bars indicate standard deviations. ns: not significant, *p<0.05, **p<0.01, ***p<0.001.
